# Current understanding of functional peptides encoded by lncRNA in cancer

**DOI:** 10.1186/s12935-024-03446-7

**Published:** 2024-07-19

**Authors:** Hua Tian, Lu Tang, Zihan Yang, Yanxi Xiang, Qi Min, Mengshuang Yin, Huili You, Zhangang Xiao, Jing Shen

**Affiliations:** 1 Laboratory of Molecular Pharmacology, Department of Pharmacology, School of Pharmacy, Southwest Medical University, Luzhou, 646000 China; 2 Cell Therapy and Cell Drugs of Luzhou Key Laboratory, Luzhou, 646000 China; 3 South Sichuan Institute of Translational Medicine, Luzhou, 646000 China; 4 School of Nursing, Chongqing College of Humanities, Science & Technology, Chongqing, China; 5 Department of Pathology, The Affiliated Hospital of Southwest Medical University, Luzhou, China 646000; 6 Gulin Traditional Chinese Medicine Hospital, Luzhou, China; 7 Department of Pharmacology, School of Pharmacy, Sichuan College of Traditional Chinese Medicine, Mianyang, China

**Keywords:** Cancer, Coding potential, Functional peptides, lncRNA, Small ORF

## Abstract

Dysregulated gene expression and imbalance of transcriptional regulation are typical features of cancer. RNA always plays a key role in these processes. Human transcripts contain many RNAs without long open reading frames (ORF, > 100 aa) and that are more than 200 bp in length. They are usually regarded as long non-coding RNA (lncRNA) which play an important role in cancer regulation, including chromatin remodeling, transcriptional regulation, translational regulation and as miRNA sponges. With the advancement of ribosome profiling and sequencing technologies, increasing research evidence revealed that some ORFs in lncRNA can also encode peptides and participate in the regulation of multiple organ tumors, which undoubtedly opens a new chapter in the field of lncRNA and oncology research. In this review, we discuss the biological function of lncRNA in tumors, the current methods to evaluate their coding potential and the role of functional small peptides encoded by lncRNA in cancers. Investigating the small peptides encoded by lncRNA and understanding the regulatory mechanisms of these functional peptides may contribute to a deeper understanding of cancer and the development of new targeted anticancer therapies.

## Introduction

Cancer threatens the health of people all over the world and is the cause of a large number of deaths each year [1]. Disorders of gene expression and imbalance in transcription are typical indicators of cancer. In fact, both coding and non-coding RNAs play a key role during these processes [2]. With the development of high-throughput sequencing technology, a large number of ncRNAs have been identified as key regulators in a variety of pathophysiological conditions including cancer [3–5]. Long non-coding RNAs (lncRNAs), by definition, refer to RNA with transcripts longer than 200 nucleotides (nt), which have no long open reading frames (ORFs, > 100 amino acids) and lack the ability to code proteins. Therefore, lncRNA were originally regarded as trash produced by the transcription process. Upon further research, lncRNAs have been proven to drive many important cancer phenotypes by interacting with other cellular macromolecules, including DNA, RNA and protein [5]. They can regulate cancer processes through chromatin remodeling [6], transcription [7] or translation [8, 9] regulation, RNA editing [10], RNA degradation [11], RNA splicing [12] and miRNA sponge [13]. Like mRNAs, lncRNAs are transcribed, spliced, capped, and polyadenylated. For a long time, it was overlooked whether the short open reading frames (sORF) in lncRNAs could be translated. Recently, with the advancement of ribosome profiling and sequencing technologies, it has been proved that the sORF in lncRNAs can encode peptides (less than 100 amino acids) or small proteins that perform some important biological functions [14–16]. According to current knowledge, the sORF, with canonical start and stop codon, go through the translation process in a way similar to that of mRNA. lncRNAs that are translated preferentially localize in the cytoplasm and their translation efficiency is similar to that of mRNAs [17].

Studies have confirmed that peptides can act as key regulators of many basic cellular processes such as development, differentiation, proliferation, splicing regulation, apoptosis and cell metabolism [18–23]. In fact, both lncRNA and polypeptides encoded by lncRNA can mediate a variety of biological functions, especially in the regulation of cancer progression. lncRNA AGPG and lncRNA AFAP1-AS1 can promote tumor progression by regulating PFKFB3-mediated glycolysis reprogramming or epigenetic inhibition of p21 expression, respectively [24, 25]. HOXB-AS3 is a peptide of 53 amino acids encoded by lncRNA HOXB-AS3 [20]. The expression of HOXB-AS3 peptide is decreased in cancer tissues. The HOXB-AS3 peptide rather than lncRNA can inhibit the growth of colorectal cancer (CRC). ASRPS is a peptide of 60 amino acids encoded by LINC00908, which is down-regulated in triple-negative breast cancer (TNBC), and can inhibit angiogenesis and exert an anti-tumor effect in TNBC [26]. The deciphering of the function of lncRNA-encoded peptides/proteins has just begun.

In this review, we describe the biological function of lncRNA in cancer, introduce the coding ability of lncRNA and the current methods to evaluate the coding potential. Finally, we discuss the most updated findings on the role of small peptides encoded by lncRNA in cancer.

## Biological function of lncRNA in *cancer*

**Fig. 1:**
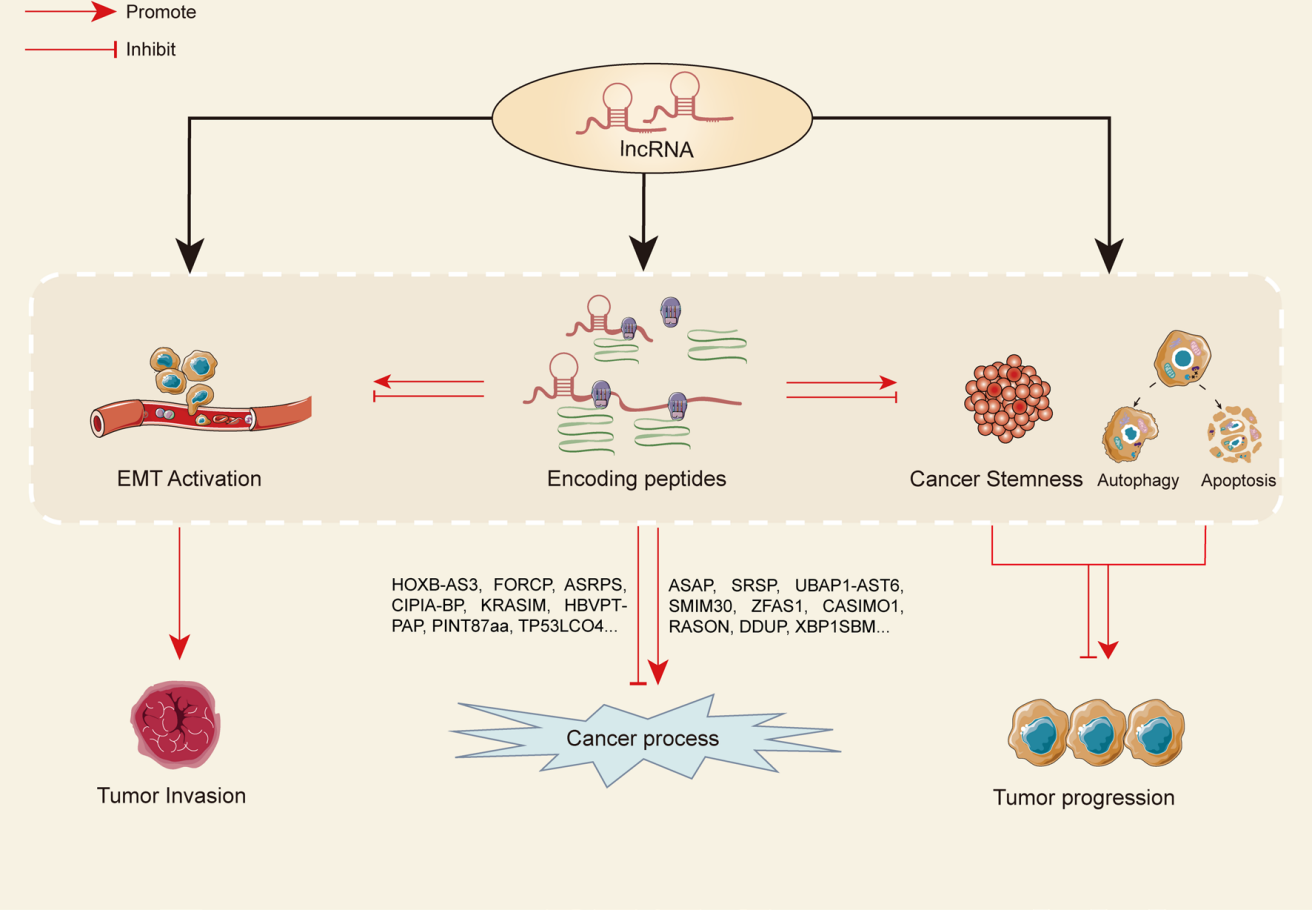
LncRNAs influence key processes in cancer. LncRNAs themselves can regulate the progression of cancer by affecting cell proliferation, migration and invasion, autophagy and apoptosis, EMT and cancer stemness. They can also encode peptides to participate in cancer regulation

LncRNA has been recognized as a key regulator in cancer [27]. Previous studies have shown that lncRNA affects some important biological functions in the tumor microenvironment including cell proliferation, migration and invasion, apoptosis and autophagy, epithelial-mesenchymal transformation (EMT), cancer stemness, etc. (Fig. 1) via mechanisms involving chromatin remodeling, RNA editing, RNA splicing, transcription and translation regulation [28–33].

### Cell proliferation, migration and invasion

Cell proliferation, migration and invasion play an important role in the progression of tumors. Through proliferation, migration and invasion, the tumor cells gradually spread to the adjacent tissue and become malignant cancers. lncRNAs are critically involved in this process. XIST (X inactivation specific transcript) is a newly discovered carcinogenic lncRNA, which promotes the malignant phenotype of many cancers [34]. Competitive endogenous RNA (ceRNA) form a network in which different RNAs, both coding and noncoding, influence each other's expression by binding to microRNA response elements (MREs). In thyroid carcinoma, XIST acts as a ceRNA sponging miR-34a and competes with MET for miR-34a binding. MET is a receptor tyrosine kinase that promotes cell proliferation through PI3K/AKT signaling and miR-34a is considered to be a tumor suppressor targeting MET, thus XIST promotes the proliferation of thyroid cancer by down-regulating miR-34a and increasing MET [35]. In pancreatic cancer (PC), ZEB1 (the key regulator of EMT and cell invasion) is the downstream target of MiR-429. Its overexpression can accelerate the migration, invasion and EMT of PC cells. XIST, as the ceRNA of miRNA-429, upregulates the expression of ZEB1, leading to PC cell migration, invasion and EMT enhancement [36]. In non-small cell lung cancer (NSCLC), XIST acts as the ceRNA of miR-744, inhibits the feedback loop of miR-744/RING1 and activates the Wnt/ β-catenin signaling pathway, which results in enhanced proliferation of NSCLC cells [37]. It has also been reported that XIST can affect tumor progression by regulating cell proliferation, migration, and invasion in nasopharyngeal carcinoma [38], glioma [39], oral squamous cell carcinoma [40], colorectal cancer [41], and hepatocellular carcinoma [42]. ABHD11-AS1 is a lncRNA that is highly expressed in various cancers including papillary thyroid carcinoma (PTC). MiR-199a-5p, as a tumor suppressor, inhibits the carcinogenic function of its downstream target SLC1A5. ABHD11-AS1, as the ceRNA of miR-199a-5p, blocks the tumor inhibitory function of miR-199a-5p, thereby enhancing the proliferation, migration and invasion of PTC cells [43]. It has also been confirmed that ABHD11-AS1 can influence tumor progression by regulating cell proliferation, migration, and invasion in colorectal cancer [44], pancreatic cancer [45], and epithelial ovarian cancer [46]. In short, the majority of cancer-related lncRNAs can affect cell proliferation, migration and invasion, thus regulating tumor progression. Some representative lncRNAs are listed in Table 1.

**Table 1:** Cancer-related lncRNA affecting cell proliferation, migration and invasion

lncRNA	Cancer type	function	Mechanism	Refer
XIST	TC	Promote proliferation	Acts as ceRNA sponge miR-34a	[35]
	PC	Promote migration, invasion and EMT	Acts as ceRNA of miRNA-429	[36]
	NSCLC	Enhanced proliferation of NSCLC cells	Acts as ceRNA of miR-744	[37]
	NPC	Promote migration, invasion	Regulating miR-30b and RECK	[38]
	Glioma	Promotes proliferation and metastasis	Regulating miR-133a/SOX4	[39]
	OSCC	Promotes proliferation & cisplatin resistance	Downregulating miR-27b-3p	[40]
	CRC	promoted CRC metastasis	Acting as a ceRNA of miR-125b-2-3p	[41]
	HCC	Inhibit proliferation and migration	Acting as miR-497-5p molecular sponge and Targeting PDCD4	[42]
	TNBC	Inhibit proliferation and EMT	Interacts with miR-454	[49]
PVT1	NPC	Promote proliferation and clone formation	Activating the KAT2A acetyltransferase and Stabilizing HIF-1α	[50]
	NSCLC	Promote proliferation, migration and invasion	Regulating miR-551b/FGFR1 Axis	[51]
	OSCC	Enhances Proliferation and Cisplatin Resistance	Regulating miR-194-5p/HIF1a Axis	[52]
	lung cancer	Facilitating proliferation and metastasis	Promoting VEGFC expression	[53]
	CRC	Promote proliferation and apoptosis	Regulating miR‑761/MAPK1 axis	[54]
	GBC	Promotes proliferation and tumor progression	Regulating the miR-143/HK2 axis	[55]
	cervical cancer	Promote proliferation and invasion	Facilitating Smad3 expression by sponging miR-140-5p	[56]
	PC	Promote proliferation and migration	Sponge miR-448	[57]
	ovarian cancer	Promotes proliferation	Binding to miR-140	[58]
	bladder cancer	Promote growth, migration, and invasion	Regulating miR-31/ CDK1	[59]
HOTAIR	cervical cancer	Facilitating proliferation and suppress apoptosis	Sponging miR-214-3p	[60]
	GC	Promote growth, migration, and invasion	acts as a ceRNA sponge mir-331-3p	[48]
	TNBC	Promote proliferation and invasion	m6A site regulates	[61]
	NSCLC	Promotes proliferation, invasion and migration	Regulating CCL22 signaling pathway	[62]
	OSCC	Promotes proliferation and migration	Targeting miR-126	[63]
	Glioma	Promote proliferation and invasion	Sponges miR-301a-3p	[64]
	BC	Promotes cancer progression	Regulating the miR-129-5p/FZD7 axis	[65]
	CCA	Inhibit apoptosis, autophagy and promote proliferation	Regulating the miR-204-5p/HMGB1 axis	[66]
	ovarian cancer	Promotes migration and proliferation	Modulating miR-222-3p/CDK19 axis	[67]
	LSCC	Promote EMT and metastasis	Modulating PI3K/ p-AKT /AKT pathway	[68]
	CRC	Promotes cancer development	Down-regulating miRNA-34a	[69]
	HCC	Mediate cancer occurrence	Inhibit miRNA-218 expression and enhancing P14 and P16 signaling	[70]
H19	Glioma	Promotes Proliferation, Migration, and Angiogenesis	Regulating Wnt5a/β-Catenin Pathway via Targeting miR-342	[71]
	Nephroblastoma	Suppress proliferation and promote apoptosis	Regulating the miR-675/TGFBI axis	[72]
	PC	Facilitating proliferation and migration	Regulating the miR-194/PFTK1 axis	[73]
	NPC	Promote proliferation and metastasis	let-7 dependent manner	[74]
	lung cancer	Promotes proliferation and metastasis	Suppressing miR-200a function	[75]
	HCC	Promote proliferation, migration and invasion	Activating CDC42/PAK1 pathway	[76]
ABHD11-AS1	PTC	Enhancing the proliferation, migration and invasion	Acts as ceRNA of miR-199a-5p/SLC1A5 axis	[43]
	CRC	Promotes CRC development	Regulation the miR-133a/SOX4 axis	[44]
	PC	Promote proliferation, migration and invasion	Regulation the PI3K-AKT pathway	[45]
AFAP1-AS1	NSCLC	Promote proliferation, migration and invasion	Epigenetically suppressing p21 expression	[25]
	GBC	Promotes proliferation and invasion		[77]
	PDAC	Promote migration and invasion		[47]
	HCC	Promotes proliferation and invasion	Activating the RhoA/rac2 signal	[78]
SNHG5	HCC	promote proliferation, cancer stemness	Regulating UPF1 and Wnt-signaling pathway	[79]
MALAT1	GC	Promote proliferation and migration	Suppressing miR-122, miR-1297, miR-22-3p, miR-202, etc	[80]
	Melanoma	Promote proliferation, migration and invasion	Downregulating miR-23a	[81]
	ovarian cancer	Promotes proliferation and metastasis	Modulating the PI3K-AKT pathway	[82]
	BC	Promotes progression and doxorubicin resistance	Modulating miR-570-3p	[83]
	CRC	Promote the development of CRC	Modulating miR-129-5p/HMGB1 axis	[84]
	HCC	Promote HCC metastasis	Regulation of peripheral vascular infiltration caused by miRNA-613	[85]
	NSCLC	Accelerating cancer progression	Modulating miR-185-5p/MDM4 axis	[86]
TUG1	ovarian cancer	Promote proliferation, invasion and stemness	Modulating miR-186-5p/ZEB1 axis	[87]
	osteosarcoma	Facilitating proliferation and Inhibit apoptosis	Modulating miR-212-3p/FOXA1 axis	[88]
	HCC	Promotes Proliferation, Migration, and Invasion	Modulating miR-29c-3p/ COL1A1 Axis	[89]
	BC	Accelerating the malignant progression of tumor	Modulating the miR-320a/FOXQ1 axis	[90]
DANCR	TSCC	Facilitating proliferation, migration, and invasion	Modulating miR-135a-5p/KLF8 axis	[91]
SNHG4	lung cancer	Facilitating proliferation, migration, invasiveness, and EMT	Modulating miR-98-5p	[92]
LUCAT1	cervical cancer	Facilitating proliferation, migration and invasion	Regulating miR-181a	[93]
FGD5-AS1	ovarian cancer	Accelerating the progression of cancer	Regulating miR-142-5p	[94]
TINCR	lung cancer	Inhibit proliferation and invasion	Modulating miR-544a/FBXW7 axis	[95]
CASC11	CRC	Accelerating the Proliferation and Migration	Sponging miR-646 and miR-381-3p	[96]
MALAT1	Melanoma	Facilitating Proliferation, Migration, and Invasion	Suppressing the expression of miR-23a	[81]
	ovarian cancer	Facilitating proliferation and metastasis	Regulating PI3K-AKT pathway	[82]
	BC	Promote the progression of cancer	Modulating miR-570-3p	[83]
ATB	Lung cancer	Facilitating Proliferation, Migration, and Invasion	Regulating microRNA-590-5p/NF90 Axis	[97]
THAP9-AS1	PDAC	Promote tumor growth	Regulating miR-484 and YAP	[98]
HOXB-AS3	Lung cancer	Facilitating proliferation, migration, and invasion	Regulating PI3K-AKT pathway	[99]

### Apoptosis and autophagy

Apoptosis (or type I cell death) is an orderly cellular process in living organisms, which features a variety of morphological changes including cell shrinkage, nuclear fragmentation and chromatin condensation. In this manner, all cellular components are eventually degraded and digested by other living cells [100]. Autophagy is characterized by the presence of autophagic vacuoles (autophagosomes) which are finally delivered to lysosomes for degradation. It often causes damaged cells or excess aging proteins/organelles to be swallowed and degraded, making the cells more conducive for survival [101]. However, in some circumstances, it also activates an alternative cell death pathway (or type II cell death) [102]. Many lncRNAs affect tumor progression by regulating these two processes. In gastric cancer (GC), lncRNA HAGLROS inhibits autophagic cell death by competitively sponging miR-100-5p to increase mTOR expression. At the same time, HAGLROS interacts and activates the mTORC1 signaling pathway, which serves as a negative regulator of autophagy, thus promoting the proliferation of GC cells [103]. LncRNA DANCR is transcriptionally activated by KLF5, a gene highly expressed in GC. Knockdown of KLF5 inhibits the DANCR/miR-194/AKT2 axis to enhance autophagy and decrease cancer cell viability [104]. LncRNA SNHG11 accelerates the progression of GC by activating oncogenic autophagy, which is dependent on the induction of ATG12 through the miR-483-3p/miR-1276 [105]. In glioblastoma, lncRNA AC003092.1 acts as a ceRNA to suppress miR-195 and promote the expression of Konitz protease inhibitor (TFPI-2). It enhances temozolomide (TMZ) chemosensitivity through TFPI-2-induced cell apoptosis [106]. It has also been reported that lncRNA CASC9 promotes cancer progression by enhancing cell proliferation and inhibiting autophagy dependent cell apoptosis by activating the AKT/mTOR signaling pathway in oral squamous cell carcinoma [107]. LncRNA NBR2 can repress autophagy-induced cell proliferation and down-regulate ERK and JNK signals in hepatocellular carcinoma (HCC), thereby inhibiting the malignant progression of HCC [108].

### Epithelial-mesenchymal transformation (EMT)

EMT refers to the transition of cells from an epithelial state to a mesenchymal state and is associated with various tumor processes such as tumorigenesis, metastasis, invasion, and malignant progression [109–111]. EMT can be divided into partial, incomplete or mixed EMT states based on the characteristics between the epithelial state and the complete mesenchymal state of the cells [112]. Some lncRNAs may play a role in the progression of cancer by affecting EMT. In NSCLC, lncRNA linc00673 has been shown to facilitate tumor progression through EMT by acting as a ceRNA to sponge miR-150-5p, leading to increased expression of ZEB1 which is a proven regulatory factor in the promotion of EMT, thus enhancing the proliferation, migration, invasion and EMT of NSCLC [113]. In HCC, MiR-15b is a carcinogenic gene that promotes cancer migration, invasion, EMT and angiogenesis. Programmed cell death 4 (PDCD4) is a tumor suppressor gene that suppresses tumor migration, invasion, EMT and angiogenesis. Interestingly, IncRNA miR503HG acts as a ceRNA to sponge miR-15b leading to increased PDCD4 expression, which in turn inhibits HCC migration, invasion, EMT and angiogenesis [114]. LncRNA AB073614 is significantly up-regulated in CRC, resulting in enhanced activation of JAK/STAT3 signaling, a pathway that promotes cancer metastasis and EMT and accelerates tumor progression [115]. In lung cancer, lncRNA-LINP1 can down-regulate transforming growth factor β (TGF- β), a key regulator of EMT in tumor progression, and inhibit tumor EMT through the TGF- β / SMAD pathway [116].

### *Cancer* stemness

Some cancer cells in the tumor cell population have the ability to self-renew, differentiate and proliferate. They resist anticancer treatments and maintain survival of cancer cells. These cells are called cancer stem cells (CSCs) [117]. Undoubtedly, CSCs have become a stumbling block in the treatment of tumors and investigation of them is deemed to hold great potential in cancer treatment. In recent years, some lncRNAs have been reported to be involved in the regulation of cancer stemness. In CRC, lncRNA H19 is expressed by cancer associated fibroblasts (CAFs) and delivered by CAF derived exosome. It acts as a ceRNA sponge of miR-141 (CRC cell stemness inhibitor) to activate β-catenin signaling and promote the stemness of CRC cells [118]. In GC, lncRNA MACC1-AS1 is induced by transforming growth factor β1 (TGF-β1) secreted by mesenchymal stem cells (MSCs), which suppresses the expression of miR-145-5p (the stemness & chemoresistance inhibitor) to accelerate FAO-dependent (fatty acid oxidation) stemness and chemotherapeutic resistance of GC cells [29]. In breast cancer (BC), lncRNA FGF13-AS1 can regulate RNA binding proteins and insulin-like growth factor 2 mRNA binding proteins (IGF2BPs) to shorten the half-life of c-Myc (Myc) mRNA. Myc (c-Myc) is a recognized carcinogenic transcription factor, which regulates cancer cell growth, proliferation, apoptosis and stemness [119]. LncRNA FGF13-AS1 suppresses the stemness of BC cells by accelerating Myc metabolism. In general, lncRNA can regulate cancer stemness, which is an important biological function of cancer-related lncRNA.

### Tumor immunity

Recent studies have found that lncRNA not only regulates the progression of many cancers, but also participates in the regulation of immune process, which plays an important role in innate immunity and acquired immunity [209]. LncRNAs may affect cancer progression through immune regulation [210]. Based on this, ImmLnc, which is a tool designed to identify immunomodulatory related lncRNA, has been developed by researchers [211].

## Ability of lncRNA to encode small peptides

As early as 20 years ago, a study revealed that lncRNA possesses sORF that can encode small peptides with biological functions in soybean [120]. After further research, it has been confirmed that these short ORFs from lncRNA can be captured by ribosomes and then translated into corresponding peptides with biological functions. In Drosophila, a ncRNA called polished rice (pri) actually has several short sORFs that encode peptides of 11 or 32 amino acids in length and participate in epithelial morphogenesis [121]. In human, as early as 2015, lncRNA CRNDE has been found to encode an endogenous CRNDEP peptide, which was located in the nucleus and may participate in oxygen metabolism and regulate cell proliferation [172]. With the in-depth study of peptides/proteins encoded by lncRNA in recent years, some lncRNAs with the ability to encode small peptides have been found. In addition, they have some biological functions, especially in tumor regulation. Interestingly, these lncRNAs can function both through their RNAs and the encoded peptides, such as lncRNA HOXB-AS3. Research has shown that lncRNA HOXB-AS3 can bind ErbB3-binding protein 1 (EBP1) and affect the EBP1-NPM1 complex formation to regulate rRNA transcription [123]. At the same time, it can also encode a 53aa peptide that plays oncogenic or tumor suppressive roles in different cancers, which will be discussed later. Overall, peptides encoded by lncRNAs can participate in the regulation of various cancer processes. This is usually achieved by binding of small peptides to their downstream targets, which are directly or indirectly involved in the occurrence and development of cancer. The binding of peptides with their targets will change the balance of tumor processes, stabilizing oncogenic signals or inhibiting tumor suppressive pathways to promote cancer progression, or upregulating tumor suppressor signals or inhibiting oncogenic pathways to inhibit cancer progression (Fig. 1).

In fact, some lncRNAs’ ORF can be translated and these small peptides are further processed into small antigenic peptides on MHC class I proteins, activating CD8 immune cells to inhibit tumor growth [212]. The peptides encoded by lncRNA can also mediate antigen presentation, CD4 + T cell response, interleukin production, etc. in the immune process [213]. This is an important functional discovery of lncRNA-derived peptides, which will drive the research of tumor immunotherapy and cancer vaccines to a new level.

## Prediction of lncRNA coding potential

With the advancement of high-throughput sequencing technology, RNA-seq can be used to identify protein-coding RNA and non-coding RNA [124]. Methods built on this have been developed to predict the coding potential of lncRNAs, using computer-based, high-throughput sequencing technology and experimental methods that comprehensively evaluate the sequence characteristics and ORF of lncRNA, and finally predict the coding potential of lncRNA and identify candidate small peptides (Table 2).

**Table 2:** Tools for predicting lncRNA coding potential

Tools	platform	Link	Functions and features	Refer
TransLnc		http://bio-bigdata.hrbmu.edu.cn/TransLnc/	Prediction of peptides encoded by lncRNA in multiple species and provide computationally predicted tumor neoantigens from peptides encoded by lncRNAs	[125]
PhyloCSF	GNU/ Linux/ Mac OS	http://compbio.mit.edu/PhyloCSF	Nucleotide sequence analysis and Protein coding region determination	[126]
COME	Linux	https://github.com/lulab/COME	Identify and characterize lncRNAs with multi-feature support	[127]
CPAT	Linux Windows	http://code.google.com/p/cpat/	Rapidly recognizes coding and noncoding transcripts	[128]
PORTRAIT	Linux	http://bioinformatics.cenargen.embrapa.br/portrait	Predicted putative proteins are evaluated for coding potential by SVM	[129]
CONC	Linux	http://cubic.bioc.columbia.edu/~liu/conc/	Evaluation of coding potential by protein characteristics	[130]
CPC	Linux	http://cpc.cbi.pku.edu.cn	Prediction based on six biologically meaningful sequence features	[131]
CPC2	Linux	http://cpc2.cbi.pku.edu.cn	More fast and accurate than CPC1, applying to ncRNA	[132]
RNAcode	Linux Windows	http://wash.github.com/rnacode	Detecting coding regions in multiple sequence alignments	[133]
sORFfinder	Web server	http://www.ncbi.nlm.nih.gov/gorf/orfig.cgi	A program package for identifying sORFs with high-coding potential	[134]
GWIPS-viz	Web server	http://gwips.ucc.ie/	a database for the identification of sORFs based on ribosome occupancy analysis	[135, 136]
MiPepid	Linux Windows	https://github.com/MindAI/MiPepid	A machine-learning tool specifically for the identification of micropeptides	[137]
NAMS webserver	Web server	http://sunlab.cpy.cuhk.edu.hk/NAMS/	Coding potential assessment and functional annotation of plant transcripts	[138]
DeepCPP	Linux Windows	https://github.com/yuuuuzhang/DeepCPP	A deep learning method for RNA coding potential prediction	[139]
SPENCER	Web server	https://spencer.renlab.org/#/home	A comprehensive database for small peptides encoded by ncRNA in cancer patients	[214]
SEP	Web server	https://ngdc.cncb.ac.cn/omix/release/OMIX266	A database that attempts to maximize a collection of SEPs from human and mouse lncRNA transcripts	[215]
FuncPEP	Web server	https://bioinformatics.mdanderson.org/Supplements/FuncPEP/	A database of Functional Peptides Encoded by Non-Coding RNAs	[216]
ncEP	Web server	http://www.jianglab.cn/ncEP/	A verified peptide database encoded by non-coding RNAs	[217]

### Methods based on sequence

Advances in sequencing technologies have led to more precise assessment of sequence features of lncRNA, including sequence homology, conservation, nucleotide composition, secondary structure, Open Reading Frame (ORF), etc. Relying on a variety of sequencing results, the coding potential of lncRNA can be predicted by methods with high precision based on sequence features. These methods include COME, CPAT and PORTRAIT. Coding Potential Computation Tool (COME) not only has high prediction accuracy, but also characterizes multiple coding parameters of lncRNA and is applicable to a variety of organisms [127]. CPAT is a tool that uses a logistic regression model to predict the coding performance of lncRNAs, and the model is based on the comprehensive evaluation of four sequence features: ORF size, ORF coverage, Fickett TESTCODE statistic and hexamer usage bias [128]. PORTRAIT is a software originally proposed to screen ncRNAs in transcriptomes from not so well-characterized organisms. Based on the support vector machine (SVM) algorithm, protein-coding or non-coding genes can be classified with high specificity. Thus it can be used to evaluate the coding potential of ncRNAs [129].

In addition, the coding potential of lncRNAs can be evaluated based on the sequence features of their ORFs. Research has found that lncRNA contain several ORFs shorter than mRNAs (sORFs), in which the longer ORFs located in the cytoplasm may be more easily captured by ribosomes, thereby facilitating translation [17, 140, 141]. We can distinguish whether the translated ORF comes from lncRNA or from mRNA via marker mapping and mutation experiments [142, 143]. Therefore, the identification of sORFs is an important indicator when evaluating the coding capacity of lncRNA. There are two important characteristics in the ORF sequence: one is the length and the other is the integrity [130, 144]. Moreover, ORFs that can be translated should have sequence homology and should be conserved. By identifying sORFs (10–100 amino acids) with coding potential in a given nucleotide sequence, a tool to identify sORFs in lncRNA was developed: sORFfinder [134]. Through this package, the ORFs from lncRNA can be calculated quickly. This kind of software is developing rapidly, and the latest version is csORF-finder designed by NUAA, which makes the computing performance more powerful [145]. Additionally, GWIPS-Viz [135, 136] is a database for the identification of sORFs based on ribosome occupancy analysis, which can identify ORFs with high coding potential in lncRNA (lncRNA has a large number of identical and conservative fragments protected by ribosomes). A peptide encoded by lncRNA has been predicted in this manner and has proven to be biologically functional [26].

### Computer-based machine learning methods

In the past decade, machine learning technology has been widely used in biological applications including genome annotation [146, 147], protein binding prediction [148, 149], identification of key transcriptional drivers of cancer [150, 151], prediction of metabolic function of complex microbial communities and characterization of transcriptional regulatory networks [152–155]. In recent years, with the development of bioinformatics, machine learning technology has been applied to the prediction of gene expression by building models [156], the study of gene splicing [157], and the identification of long non-coding RNA [158–161]. Even more recently, machine learning methods have been applied to the identification of transcript coding potential. After the discovery that some non-coding RNAs have the ability to encode peptides, the machine learning technology was brought to the prediction of the coding ability of ncRNA. MiPepid [137], a tool dedicated to the identification of micropeptides, is based on machine learning techniques. MiPepid has been trained using an existing database and logistic regression with 4-mer features to achieve a high degree of accuracy while running fast. DeepCPP [139], a deep neural network for RNA coding potential prediction, is based on nucleotide bias information and minimum distribution similarity features. Its advantage is the improved ability to identify sORFs RNA (lncRNA, etc.). NAMS webserver [138] is a web server that predicts the coding potential and functional annotation of plant transcripts. Based on its computing power, it can also be used to evaluate the coding potential of lncRNA genes.

### Experimental approach

As more and more functional small peptides encoded by lncRNA are discovered, researchers have also designed experimental methods for verification. Ribosomal sequencing is a method used to evaluate the coding potential of ncRNAs. By sequencing ribosome-protected RNA fragments, the ribosome enrichment information of lncRNA sequences can be used to predict whether the lncRNA has the potential to encode peptides [17]. Many lncRNA encoded peptides have been discovered in this manner [20, 162]. After the initial identification, a vector expressing the FLAG-labeled ORF can be transfected into cells. If the ORF is translatable, it will drive the FLAG tag to be translated together, and immunofluorescence can be used to determine whether the corresponding protein is produced followed by other methodologies such as Western Blot, etc. [163–166]. Next, the start codon can be mutated, and once again used to detect whether there is a FLAG band at the corresponding molecular weight by Western blotting, in order to determine whether the translation occurs normally [167]. At the same time, the endogenously expressed lncRNA encoded peptide can be detected with antibodies raised against amino acid sequences in the peptide by western blotting and/or IP/MS. In addition, pull-down experiments such as co-immunoprecipitation (CO-IP) can also be used to discover proteins that interact with the functional peptide [168], which can further be detected by MS.

## Functions of peptides encoded by LncRNA in *cancer*

**Fig. 2:**
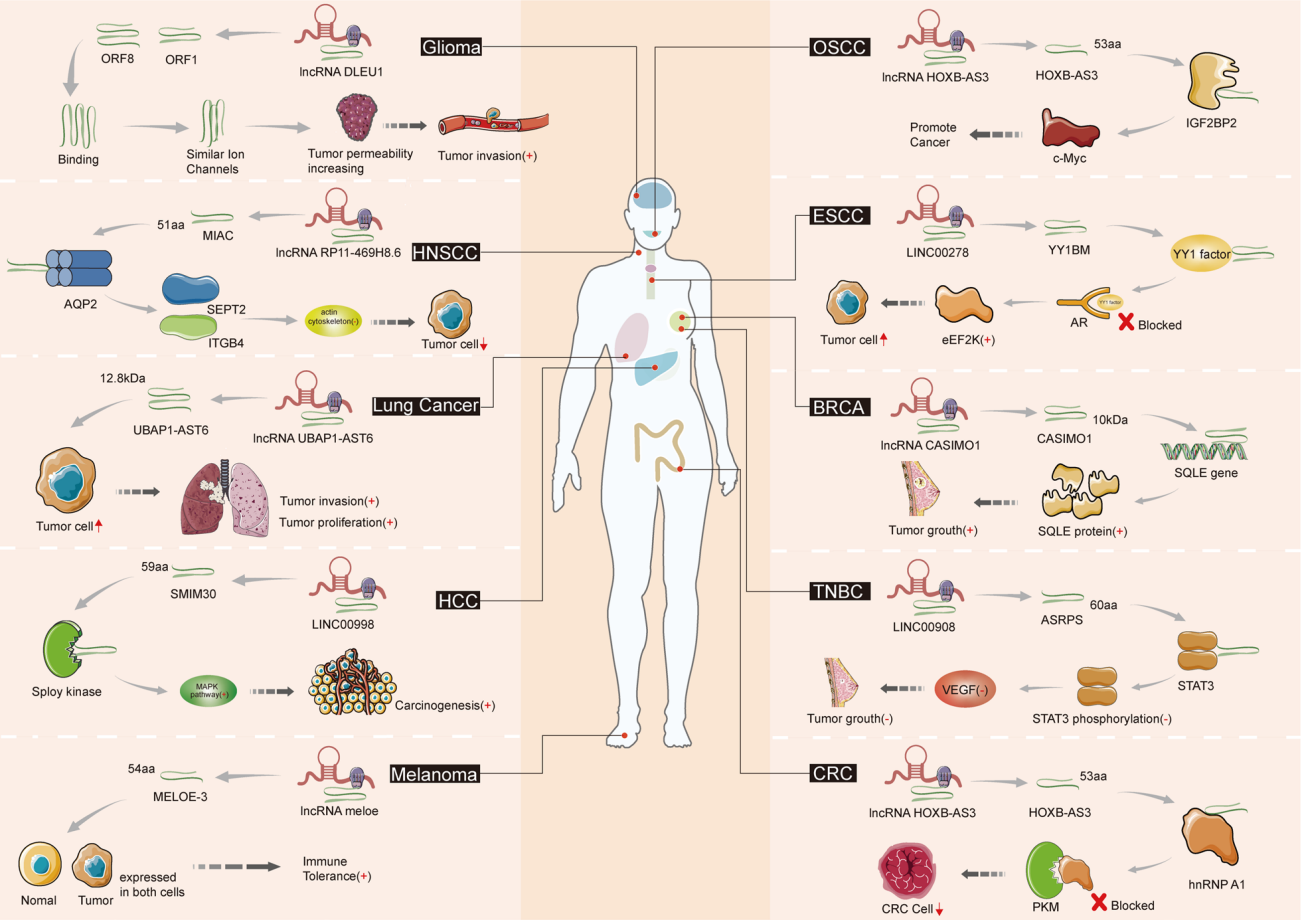
Functions of peptides encoded by lncRNA in cancer. LncRNA can encode peptides to promote tumor development in glioma, lung cancer, HCC, OSCC, ESCC, breast cancer and melanoma or inhibit tumor development in HNSCC, TNBC and CRC

Some lncRNAs possess small open reading frames (sORFs), which can exert biological functions by encoding functional small peptides. Several studies have found that small peptides encoded by lncRNA play a key role in the regulation of various cancer processes [169]. Some small peptides can cause worsening of the cancer phenotype and manifest as oncogenic peptides, while others can inhibit tumor proliferation, metastasis and invasion, manifested as tumor suppressor peptides. RNA-binding regulatory peptide (RBRP) is a functional peptide of 71aa encoded by lncRNA LINC00266-1. Studies have shown that RBRP is a regulatory subunit of RNA m^6^A reader IGF2BP1 complex by binding directly to the GxxG motif in the KH3–4 di-domain of IGF2BP1, which is indispensable for m^6^A recognition and interaction. The increased expression of RBRP in tumors promotes the recognition and binding of m^6^A reader IGF2BP1 to the targeted RNA in order to enhance the mRNA stability of proto-oncogenes (such as c-Myc, etc.), which induces tumorigenesis [170]. PACMP is a 44aa micropeptide encoded by lncRNA CTD-2256P15.2 with multiple functions. It not only suppresses CtIP-KLHL15-dependent CtIP ubiquitination, but also promotes PARP1-induced PAR polymerization by combining with DNA damage-mediated polychains. Both PARP1 and CtIP are important targets in cancer, so PACMP has the potential to become a high-value anti-cancer target [122]. The forced expression of TUBL, a 87aa peptide encoded by lncRNA TINCR, promotes cell cycle progression in normal human epidermal keratinocytes. Mice lacking this protein exhibit decreased cell cycle progression in skin-keratinocytes, delayed wound healing, and the protein may promote the proliferation of cancer cells [171]. A similar functional peptide encoded by lncRNA that is involved in cell proliferation is CRNDEP (84aa) encoded by lncRNA CRNDE [172]. A number of functional small peptides encoded by lncRNAs play crucial roles in different types of cancers (Table 3 and Fig. 2).

**Table 3:** Peptides encoded by lncRNA in cancer

Cancer type	Pep name	Pep length	lncRNA	Function	Mechanism	Refer
Multi Cancer	RBRP	71aa	LINC00266-1	tumorigenesis	Binding to IGF2BP1	[170]
	TUBL	87aa	TINCR	cell proliferation		[171]
	PACMP	44aa	CTD-2256P15.2	Regulate drug resistance	Inhibit the degradation of CtIP by KLHL15 and promote PARP1-dependent PARylation	[122]
Hela cell	CRNDEP	84aa	CRNDE			[172]
CRC	HOXB-AS3	53-aa	lncRNA HOXB-AS3	Suppress CRC growth	Binds to arginine residues and inhibits reprogramming of glucose metabolism	[20]
	pep-AP		lnc-AP	Chemosensitive	Interacts with TALDO1 protein to inhibit its expression	[173]
	ASAP	94aa	LINC00467	Promote CA progression	Interacts with ATP5A and ATP5C to promote CRC progression	[174]
	PVT1		lncRNA PVT1	Immune Surveillance	Recognized by CD8 tumor-infiltrating lymphocytes and mononuclear cells	[175]
	FORCP	79 aa	LINC00675	Inhibit tumorigenesis	FORCP depletion results in decreased apoptosis	[176]
	SRSP	130aa	LOC90024	promotes CRC progression	Induces "cancerous" Sp4 splicing variant formation	[177]
	UBAP1-AST6	12.8 kDa	LncRNA UBAP1-AST6	Promote CA progression		[178]
TNBC	ASRPS	60-aa	LINC00908	Inhibit tumor angiogenesis	ASRPS directly bound to STAT3 and down-regulated STAT3 phosphorylation	[26]
	CIP2A-BP	5.5KDa	LINC00665	Inhibited tumor progression	Binds tumor oncogenes to inhibit PI3K/AKT/NFκB pathway	[179]
	XBP1SBM	21aa	lncRNA MLLT4-AS1	promotes angiogenesis and metastasis	Promote the expression of VEGF	[180]
HCC	SMIM30	59aa	LINC00998	promotes HCC development	Activates the MAPK signaling pathway	[181]
	KRASIM	99-aa	lncRNA NCBP2-AS2	suppress HCC growth	Binds KRAS protein to inhibit ERK signaling	[182]
	HBVPTPAP	145 aa	lncRNA HBVPTPAP	Inducing apoptosis of HCC cells	Activates the JAK/STAT pathway by interacting with PILRA	[183]
	PINT87aa	87aa	LINC-PINT	anti-proliferation in HCC cells	Binds to FOXM1 to block PHB2 transcription	[184]
	ZFAS1		lncRNA ZFAS1	Promotes CA Cell Migration	Inhibiting nicotinamide adenine dinucleotide dehydrogenase expression	[185]
	TP53LC04	100aa	lncRNA AC022075.1	Inhibit cell proliferation	Regulation of cell cycle and DNA damage	[186]
Glioma	ORF1/ ORF8		DLEU1	promotes CA progression	encode small peptides with ion channel activity	[187]
OSCC	HOXB-AS3	53-aa	lncRNA HOXB-AS3	Promote CA progression	Binds to IGF2BP2 to maintain the stability of c-Myc mRNA	[188]
Lung cancer	UBAP1-AST6	12.8 kDa	LncRNA UBAP1-AST6	promote tumor progression	significantly promote cell proliferation and clone formation	[178]
Melanoma	MELOE-3	54 aa	LncRNA meloe	Produce immune tolerance	result from its expression in normal melanocytes	[189]
Breast cancer	CASIMO1	10 kDa	lncRNA CASIMO1	Promote tumor progression	interact with members of the mevalonate (MVA) pathway	[190]
ESCC	YY1BM	21 aa	LINC00278	Involved in the ESCC progression	YY1BM blocked YY1 binding to AR to activate the expression of eEF2K	[191]
PDAC	RASON	108aa	LINC00673	promotes CA progression	Promote the expression of carcinogenic RAS pathway	[192]
ovarian cancer	DDUP	186aa	lncRNA CTBP1	Promote drug resistance to chemotherapy	DNA damage repair	[193]
HNSCC	MIAC	51 aa	LncRNA RP11-469H8.6	Inhibits HNSCC Progression	directly interacts with AQP2 (Aquaporin 2) to inhibit the actin cytoskeleton	[194]

### Hepatocellular carcinoma (HCC)

Liver cancer ranks fourth among all cancer-related deaths worldwide, and a large number of people die from hepatocellular carcinoma (HCC) each year [195]. It has been confirmed that a variety of small proteins encoded by lncRNA can affect the occurrence and progression of HCC. SMIM30 is a 59aa peptide encoded by LINC00998. SMIM30 promotes the carcinogenesis of HCC by activating the MAPK signaling pathway. The underlying mechanism involves the binding of the peptide to non-receptor tyrosine kinase Sploy, which drives its membrane anchoring and phosphorylation, and then activates the mitogen-activated protein kinase (MAPK) pathway thus promoting the proliferation and migration of HCC cells [181]. KRASIM is a 99aa peptide encoded by lncRNA NCBP2-AS2. Overexpression of KRASIM can reduce the level of proto-oncogene KRAS protein, which then inhibits the ERK signaling pathway in HCC cells causing a reduction in the growth and proliferation of HCC cells. In the cytoplasm of human HCC cells, KRASIM interacts and colocalizes with KRAS protein [182]. The lncRNA HBVPTPAP encodes a peptide of 145aa called HBVPTPAP. HBVPTPAP is mainly located in the cytoplasm and can induce mitochondrial apoptosis by activating the JAK/STAT signaling pathway, thus inducing apoptosis of HCC cells. This regulation was through the interaction between the HBVPTPAP polypeptide and PILRA [183]. PINT87aa is a functional small peptide of 87aa encoded by LINC-PINT. PINT87aa can induce senescence of hepatoma cells by blocking PHB2 transcription through the direct binding of PINT87aa and FOXM1 DNA binding domain [184]. A study by Guo et al. has shown that lncRNA ZFAS1 has several small open reading frames (smORF), one of which has been proven to be up-regulated in HCC tumor tissue, but is scarcely expressed in normal liver tissue, which implies that smORF may be related to the occurrence and development of hepatocellular carcinoma. However, the detailed mechanism needs to be further elucidated [185].

### Colorectal *cancer* (CRC)

More than 1.2 million patients are diagnosed with colorectal cancer (CRC) each year, and more than 600,000 people die from the disease [196]. In CRC, functional small peptides encoded by a variety of lncRNAs regulate tumor progression. As mentioned above, HOXB-AS3 peptide promotes the proliferation of oral squamous cell carcinoma cells. The oncogenic role of LncRNA HOXB-AS3 has also been implicated in different cancer types. On the contrary, in CRC, lncRNA HOXB-AS3 inhibits the reprogramming of glucose metabolism through its encoded 53aa peptide. This antagonizes the regulation of splicing of pyruvate kinase M (PKM) mediated by hnRNP A1, and ultimately inhibits the growth of CRC. This process is achieved by the competitive binding of HOXB-AS3 peptide to the ariginine residues of hnRNP A1 which blocks the binding of its own arginine residue to exon 9 of PKM [20]. Further investigation is pending into whether the function of lncRNA HOXB-AS3 and its peptide differs depending on cancer type. ASAP is a 94aa peptide encoded by LINC00467, which is associated with ATP synthesis. Clinically, ASAP is associated with the malignant phenotype in patients, whereby high expression of ASAP indicates poor survival in patients with CRC. As for the mechanism, ASAP peptide promotes the proliferation of CRC cells by promoting ATP synthesis, thereby increasing the activity of ATP synthase and the rate of oxygen consumption by mitochondria, while the deletion of ASAP can inhibit the growth of colon tumors in vitro [174]. LINC00675 encodes a small protein of 79aa, FORCP, endogenously expressed mainly in the cytoplasm and can promote tumor cell apoptosis in response to stress in the endoplasmic reticulum. Functionally, FORCP can also inhibit cancer cell proliferation and clone formation to further inhibit tumor progression [176]. SRSP is a functional small peptideof 130aa that is encoded by LOC90024, which can promote CRC tumorigenesis and progression. Long SP4 isomer (L-SP4 protein) is a carcinogenic protein, while short SP4 isomer (S-SP4 peptide) is a non-carcinogenic protein. SRSP peptide increases the probability of SRSF3 binding to exon 3 of transcription factor SP4, thus inducing the formation of carcinogenic protein L-SP4, which leads to tumorigenesis [177]. Pep-AP is a short peptide encoded by lnc-AP, which can reverse the resistance of colon cancer cells and make them more sensitive to oxaliplatin [173]. Kikuchi et al. reported that PVT1 is a carcinogenic peptide encoded by lncRNA PVT1, located downstream of transcription factor *MYC* gene and is abnormally overexpressed in various cancers. In CRC, PVT1 carcinogenic peptide can be detected in CD8 T cells and peripheral blood mononuclear cells of patients [175].

### Breast *cancer* & triple-negative breast *cancer* (TNBC)

According to the global cancer statistics in 2020, breast cancer ranked first in the incidence of malignant tumors in the world, and it was the fifth leading cause of death among malignant tumors [197]. It has been reported that lncRNA CASIMO1 encodes a 10 kDa microprotein CASIMO1 in breast cancer. The overexpression of CASIMO1 causes binding to the oncogene SQLE, promoting its accumulation at the protein level and subsequently accelerates the proliferation of breast cancer. Mutation of lncRNA CASIMO1 translation promoter or knockout of lncRNA CASIMO1 causes a loss of its carcinogenic effect [190]. Triple-negative breast cancer (TNBC) accounts for 15% of breast cancers with a high degree of malignancy and usually poor prognosis. In TNBC, the three main breast cancer tumor markers: estrogen receptor (ER), progesterone receptor (PR), and Her2 are all negative [198]. ASRPS and CIP2A-BP are two small proteins with tumor suppressive roles in TNBC. ASRPS is a functional small peptide of 60aa encoded by LINC00908. ASRPS can inhibit tumor progression and the mechanism is that the ASRPS peptide binds STAT3 directly through the coiled coil domain (CCD), and then down-regulates the phosphorylation of STAT3, resulting in a decrease in the expression of VEGF [26]. A 5.5 kDa peptide encoded by LINC00665 has been designated as CIP2A-BP. CIP2A-BP can inhibit the progression of TNBC. It replaces the B56γ subunit after binding to the tumor gene CIP2A, and then stimulates PP2A activity, thus reducing the expression of MMP-2, MMP-9, and Snail by inhibiting the PI3K/AKT/NFκB signaling pathway [179].

### Upper gastrointestinal *cancer*

The oral cavity is the starting point of the upper digestive tract and has important physiological functions. Oral cancer is becoming a global public health problem. At present, about 377,000 people suffer from oral squamous cell carcinoma and 177,000 people die each year [197]. The tumor promoting role of lncRNA HOXB-AS3 has been well documented in various types of cancers [99, 199, 200]. HOXB-AS3 peptide is a small 53aa peptide encoded by lncRNA HOXB-AS3. In oral squamous cell carcinoma (OSCC), HOXB-AS3 and its encoded peptides can promote the proliferation of cancer cells, which is achieved by the direct binding of HOXB-AS3 peptide to IGF2BP2 to maintain the stability of mRNA stability of the oncogene c-Myc [188]. Esophageal squamous cell carcinoma (ESCC) is threatening more than 400,000 people worldwide, and men are more vulnerable than women [197]. A study by Wu *et a.l* has shown that LINC00278, a Y-linked lncRNA, down-regulated in male ESCC, encoded a micropeptide called YY1BM. YY1BM binds to YY1 to suppress the expression of eEF2K simulated by YY1 and the androgen receptor (AR), which promotes apoptosis and inhibits the proliferation of ESCC. However, YY1BM is down-regulated in ESCC, which in turn promotes cancer progression [191].

### Other types of *cancer*

Lung cancer is especially important in the eyes of the public, since it causes more than 1.76 million death per year [201]. Studies with lung cancer have found that lncRNA UBAP1-AST6 encodes a peptide of 12.8 kDa, called UBAP1-AST6. It can promote cell proliferation and clone formation, but the specific mechanism is not clear. This peptide may promote the occurrence and development of tumors [178]. Pancreatic ductal carcinoma (PDAC) is a malignant tumor with low survival rate. The vast majority of PDAC patients have KRAS mutations [202]. Rason is a 108aa peptide encoded by LINC00673. Rason prolongs the active state of KRAS signaling by binding to KRAS in order to drive tumorigenesis and metastasis [192]. Melanoma is a type of skin cancer caused by melanocytes, the pigment-producing cells found in tissues such as the epidermis, hair follicles, and the iris. In most countries, the incidence of melanoma has been increasing over the past few decades [203, 204]. Delphine *et a.l* found two new polypeptides, MELOE-1 and MELOE-2, which are involved in immunosurveillance [205]. The same researchers also discovered MELOE-3, a 54aa functional small peptide encoded by lncRNA meloe, which is expressed in both melanoma cells and normal melanocytes. MELOE-3 has poor immunogenicity in melanoma cells, and the protein expressed in the physiological state is also associated with immune tolerance [189]. This provides a promising T cell target for melanoma immunotherapy.

In addition to the above cancers, peptides encoded by lncRNA have been identified to drive the progression of other cancers, including head and neck squamous cell carcinoma (HNSCC), glioma and ovarian cancer. Head and neck squamous cell carcinoma (HNSCC) is the sixth most common cancer globally with a high mortality rate of 40 to 50% [206]. MIAC is the first micropeptide, 51aa in length, found in head and neck squamous cell carcinoma, which is encoded by lncRNA RP11-469H8.6. MIAC directly binds to aquaporin 2 (AQP2) to suppress the expression of SEPT2 & ITGB4. It then inhibits the actin cytoskeleton, which is a key regulatory factor in the migration and invasion of cancer cells, thus suppressing tumor growth and metastasis [194]. Glioma is the most common primary tumor in the brain, accounting for up to 81% of malignant brain tumors. Although relatively rare, it has a high mortality rate [207]. Cao et al. predicted lncRNAs which may encode small transmembrane peptides in gliomas using in silico approaches. They demonstrated that lncRNA DLEU1 has two smORFs (ORF1 and ORF8). DLEU1 encodes small peptides, ORF1 and ORF8, which can aggregate to form similar ion channels and lead to an increase in the permeability of the glioma [187]. However, the expression and function of these peptides need to be verified by further experiments. Ovarian cancer is a type of tumor among females which has a high degree of malignancy. It has a low survival rate and threatens the health of most women [208]. DDUP is a polypeptide of 186aa encoded by lncRNA CTBP1. In ovarian cancer, DDUP can bind to ATR kinase activated by DNA damage and can be phosphorylated, resulting in structural changes in DDUP. Conformational changes aggravate the binding ability of DDUP to rapidly phosphorylated histone H2AX (γ-H2AX) and RAD18 (transduction of DNA damage signal), resulting in the formation of a stable γ-H2AX/DDUP/RAD18 complex and persistent retention of RAD18 foci at the injured site [193]. This leads to resistance to chemotherapy and radiotherapy based on DNA damage.

## Conclusions and future perspectives

Recent studies have shown that some originally defined lncRNAs can participate in the regulation of multi-organ tumors by encoding functional small peptides, which totally changed our understanding of these supposedly non-coding RNAs. Functional peptides, such as HOXB-AS3, pep-AP, ASAP, PVT1, SRSP, etc. are encoded by lncRNAs and promote or inhibit the development of CRC through a series of regulatory processes. In other cancers, there are similar small peptides/proteins, which are encoded by lncRNAs and affect the occurrence and development of tumors. Research in this area has just begun and these new findings may lead to change in the classification of these lncRNAs in the future.

At present, it has been confirmed that lncRNAs can regulate the occurrence and development of tumors either by themselves or through encoded small peptides. Although this has great application value, such as the development of new antineoplastic drugs and/or new cancer biomarkers, deeper research is needed before the real clinical applications are possible. At the current stage, several questions remain to be answered. First, until now, studies have only identified a small number of lncRNA-encoded peptides. There are still an inestimable number of functional peptides encoded by lncRNA waiting to be discovered. Over time, we will get a clearer picture of the role of these peptides and will be able to determine whether they are evolutionarily important. Second, with the development of technology, more advanced tools are needed to accurately predict and validate the coding potential of lncRNAs. Third, the mechanism by which many small peptides affect tumor progression is not clear. It remains unclear as to whether the small peptides themselves or lncRNAs or both are functional. Scientists need to carefully discriminate between the functions of lnRNA and that of the encoded peptides or to determine whether the lncRNA is bifunctional. Finally, if these micropeptides are going to be used as potential anti-tumor targets, the upstream regulatory mechanism and downstream binding proteins need to be further clarified. Despite these problems, lncRNA-encoded peptides are a promising resource for the development of new diagnostic and prognostic biomarkers and/or therapeutic targets in cancer, which merits further and an in-depth investigation. It will be a widely-studied topic in cancer research in years to come and will undoubtedly push research into human biomedicine to a new level.

## Data Availability

Not applicable.
